# Multi-omics model is an effective means to diagnose benign and malignant pulmonary nodules

**DOI:** 10.1016/j.clinsp.2025.100599

**Published:** 2025-02-21

**Authors:** Yunzeng Zhang, Fan Zhang, Changming Shen, Gaofeng Qiao, Cheng Wang, Feng Jin, Xiaogang Zhao

**Affiliations:** a Department of Thoracic Surgery, Shandong Public Health Clinical Center, Shandong University, Shandong, China; b Department of Thoracic Surgery, The Second Hospital of Shandong University, Jinan, Shandong, China; c Provincial Key Laboratory for Respiratory Infectious Diseases in Shandong, Shandong University, Shandong, China

**Keywords:** Multi-omics model, Diagnose, Benign, Malignant, Pulmonary nodules

## Abstract

In response to the high false positive rate of traditional Low-Dose Computed Tomography (LDCT) in diagnosing pulmonary malignant nodules, this study aimed to investigate the effectiveness of scoring of blood-based non-invasive biological metabolite detection combined with artificial intelligent scoring of non-invasive imaging in the clinical diagnosis of Pulmonary Nodules (PNs). In this retrospective study, risk scoring was performed in patients positive for pulmonary nodules and subsequently, PNs were sampled by invasive procedures for pathological examinations. The pathological classification was used as the gold standard, and statistical and machine learning methods showed, that in 210 patients (23 benign PN and 187 malignant PN), the risk score of Metabonomics, radiomics, and multi-omics had different levels of performance in different risk groups based on various predictive models. The Area Under the receiver operating Characteristic Curve (AUC) of the multi-omics model was 0.823. The present results indicate that a multi-omics model is more effective than a single model in the non-invasive diagnosis of pulmonary malignant nodules.

## Highlights

• Multi-omics study is promising to improve the sensitivity and specificity of screening for NSCLC.
• The risk score of Metabonomics, radiomics and multi-omics had different levels of performance in different risk groups.
• Multi-omics model is more effective in the non-invasive diagnosis of pulmonary malignant nodules.

## Background

Lung cancer is a malignant tumor with the highest incidence and mortality in China and has been the greatest threat to human health and life.^1^ Due to the insidious early symptoms, 60 %‒80 % of lung cancer is often diagnosed at the advanced stage, and thus the 5-year survival rate of lung cancer patients is only about 5.6 %..^2^ Therefore, early diagnosis and treatment of lung cancer is an effective way to reduce the mortality of lung cancer and improve the survival of lung cancer patients.

Currently, Low Dose Computed Tomography (LDCT) is the most widely used tool for the screening of lung cancer. However, for patients with small pulmonary nodules, distinguishing between malignant and benign nodules by using Computed Tomography (CT) has been a challenge in clinical practice, as radiological features are indistinguishable between malignant and benign nodules.^3^ To date, some biomarkers have been employed for the diagnosis of tumors, but most of these proteins are also present in the serum of cancer-free patients, which limits their clinical application in the early diagnosis of cancers.^4^ Some studies have reported that small molecule substances such as free amino acids, free fatty acids, and phospholipids in the serum exhibit significant differences between cancer patients and healthy controls, and serum is preferred for metabolomics studies.^5^^,^^6^ However, many biomarkers that are widely used to assess the risk of cancers usually have a low specificity.^7^ Only a few studies report the use of serum markers in the screening and diagnosis of cancers,^8^, ^9^, ^10^ and studies on these markers are still ongoing. Thus, clinical studies are facing challenges in the real world, and the results show significant differences among various studies. Silverstri et al.^11^ developed a model that could distinguish benign and malignant pulmonary nodules based on protein biomarkers and clinical characteristics of patients, with a sensitivity of 97 %. Therefore, the comprehensive analysis based on multi-omics studies is promising to improve the sensitivity and specificity of screening for non-small cell lung cancer, which may be helpful for clinical decision-making.

This study aimed to investigate the differentiation between malignant and benign pulmonary nodules by using multiple omics models. Studies have confirmed that Artificial Intelligence (AI) and liquid biopsy play significant roles in cancer diagnosis.^12^, ^13^, ^14^ Liquid biopsy has the advantage of avoiding interference of heterogeneous cells, and liquid biopsy with small molecules such as in peripheral blood as detection targets has gradually become a complementary solution to imaging examination. Metabolomics may more directly reflect the physiological and pathological conditions of organisms in tumor studies, which provides a new perspective for the investigations of disease biomarkers.^15^ In this study, results showed metabolic abnormalities in early non-small cell lung cancer. By using Support Vector Machine (SVM) and Mass Spectrometry (MS), serum metabonomics analysis and feature selection were performed to establish a metabolic fingerprint, and this method was confirmed to improve the specificity of detection, which helps the decision-making of clinicians.

## Materials and methods

In this study, statistical analysis and machine learning methods were employed to investigate various clinical and molecular features, aiming to identify potential biomarkers with predictive significance for malignant pulmonary nodules. Then, a prediction model was constructed and optimized. Furthermore, this optimized model was tested for validation, aiming to evaluate its generalization ability on unknown samples. The present study tends to analyze the results, their explanations, and their practical significance in clinical settings. The study follows the STROBE Statement.

In this study, lung tissues and plasma were collected from asymptomatic patients with pulmonary nodules on CT tissue. These patients subsequently underwent surgical resection from February 17, 2019, to December 10, 2019. All the patients had no history of cancer. The blood was collected before surgery in this study. This was a retrospective study, and the study procedures did not influence clinical decision-making. In the initial screening of biomarkers, patients did not receive screening or examinations before enrollment, aiming to ensure the authenticity of the cohort. In the subsequent validation, patients who received screening with LDCT were encouraged to participate in the investigation. Pathological examination was performed based on the WHO pathological classification of lung cancer (2015). The pulmonary nodules were pathologically examined in surgically obtained sections. All the study procedures were approved by the Ethics Committee of the hospital, and informed consent was obtained from each subject (2021XKYYEC-14).

### Selection of patients

Before screening, the general population aged 18 to 74 was evaluated because they are often considered to benefit from cancer screening.^16^ The inclusion criteria were as follows: 1) Chest CT showed pulmonary nodules smaller than 3 cm; 2) Participants were aged 18‒74 years and asymptomatic. The exclusion criteria are: 1) Patients had a history of lung surgery; 2) Patients had a prior history of malignant tumors; 3) The medical record was missing or incomplete. Sample collection is a very important step, and the quality of collected samples significantly affects the results. Yin et al.^17^ provided a strict experimental process and reported that hemolysis could cause changes in the metabolic spectrum. In this study, the sample quality was strictly controlled.

In this study, an AI engine for lung nodule screening was used to identify nodules in the lung based on DICOM data of chest CT images. By analyzing different specific parameters, these parameters are added to the objective function in a certain form, and then the identified nodules are interpreted, and the malignant risk of lung nodules is scored.

In the present study, 76 patients were initially diagnosed with lung cancer and did not receive any treatment and 203 healthy controls were included, and serum samples were harvested. Support Vector Machine (SVM) and integrated learning method were used to construct the metabolic fingerprinting for the diagnosis and classification of lung cancer, aiming to investigate the value of metabolomics in the differentiation of benign and malignant pulmonary nodules. Before surgery, serum biomarkers were detected by Next Generation Sequencing (NGS). Statistical analysis and machine learning were employed to screen molecular markers related to malignancy and a model was then constructed. All the data are input into the Compound Discoverer 3.1 for screening. The least squares method was used to smooth the data, and data meeting the requirements of quality control were included in the cancer classification model for risk scoring. Then, the metabolic molecules in the blood were detected and modeled.

Finally, a new scoring method was established by combining the risk score based on metabolic fingerprint with the risk score of malignancy based on artificial analysis of lung CT images. For the patients who received surgical treatment, pathological examination of pulmonary nodules was performed, and pathological results served as the gold standard.

### Statistical analysis

Continuous data are expressed as mean ± SD for normally distributed variables and medians with 25th and 75th percentiles for abnormally distributed variables. The difference between the two groups was examined using the Mann-Whitney test (non-normal distribution). Clinical features were analyzed by multiple logistic regression. All statistical analyses were performed by *R* software, and data were produced by GraphPad Prism 8 software; *p* < 0.05 was considered statistically significant.

## Results

**Fig. 1:**
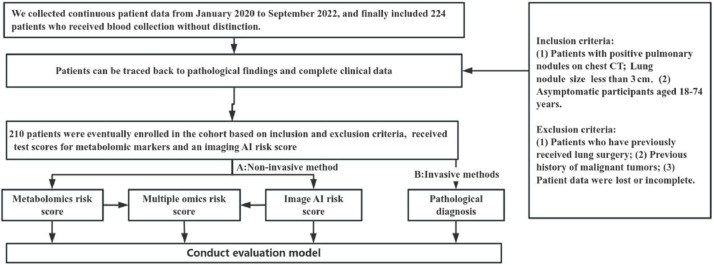
The overview of this study.

**Fig. 2:**
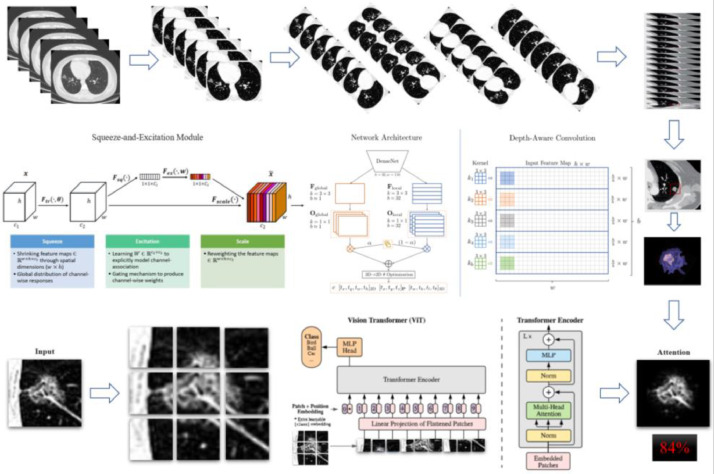
Artificial Intelligence (AI) image engines augment the original data hundreds of times to achieve shape recognition. In this study, the authors collected features to achieve a risk score for radiomics.

In the screening of biomarkers, plasma samples and CT images were collected from 224 patients. Patients were included according to the inclusion and exclusion criteria, and the clinical characteristics were obtained. Finally, samples (benign nodule: n = 23; malignant nodule: n = 187) were collected from a total of 210 patients. Multiple tests were conducted, and they were finally included in the independent validation. Fig. 1 shows the flowchart of the present study. Fig. 2 illustrates the basic principles of imagomics.

According to the final pathological results, pulmonary nodules were divided into Malignant group (MPN) and the Benign group (BPN). The clinical information of patients included age, gender, nodule length, and nodule density on CT. In 210 samples, 50.3 % of patients with MPN and 52.2 % of patients with BPN were males. In the MPN group and BPN group, the median age was 58-years and 55-years, respectively. In the MPN group and BPN group, the median number of nodules was 19 and 16, respectively. In the MPN group and BPN group, the percentage of solid nodules was 33.2 and 65.2, respectively. In the MPN group and BPN group, smokers accounted for 34.2 and 13.0, respectively. Patients were scored based on the known lung cancer model and AI analysis of identified biochemical molecules: the higher the score, the higher the risk was. The integrated scoring was based on above mentioned methods. Table 1 displays the patient cohort and their clinical characteristics.

**Table 1:** Summary of patient demographics and clinical profiles of 210 patients in this study.

		Benign (*n* = 23)	Malignant (*n* = 187)
**Age (yr, IQR)**		55 (43‒65)	58 (50‒67)
**Gender, n (%)**	Male	12 (52.2)	94 (50.3)
**Size (mm, IQR)**		16 (10‒19)	19 (16‒21)
**Smoker, n (%)**	Yes	3 (13.0)	64 (34.2)
**Solid, n (%)**	Yes	15 (65.2)	62 (33.2)
**Type, n (%)**	Squamous cell carcinoma		18 (9.6)
	Adenocarcinoma		152 (81.3)
	Others		15 (8.0)
	Small cell carcinoma		2 (1.1)
	Pulmonary tuberculosis	9 (39.1)	
	Hamartoma	1 (4.4)	
	Sclerosing Pulmonary cell tumor	1 (4.4)	
	Granulomatous inflammation	3 (13.0)	
	Atypical adenomatous hyperplasia	9 (39.1)	
**Metabonomics (core, IQR)**		67.8 (59.3‒72.0)	71.5 (70.0‒73.6)
**Radiomics (core, IQR)**		79.0 (26.0‒83.0)	81.0 (78.0‒85.0)
**Multi-omics (core, IQR)**		66.3 (58.3‒72.3)	73.5 (71.8‒75.3)

**Fig. 3:**
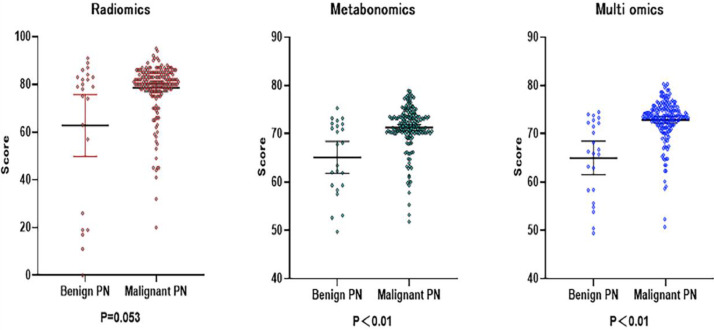
The scores for the two sets of data in the present study are shown in the figure, and the rank sum test is used between the two groups. (The higher the score, the higher the risk of having a malignant pulmonary nodule).

**Fig. 4:**
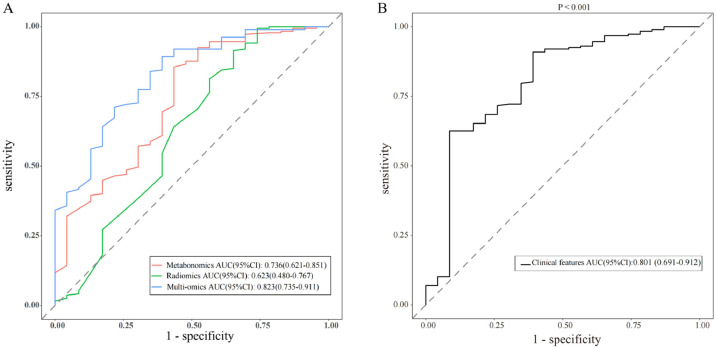
Performance of various predictive models in forms of Receiver Operating Characteristic (ROC) curves and Area Under Curve (AUC) scores (AUC and the 95 % CI of each model are shown in the legend). (A) Shows the diagnostic model of metabonomic, radiomics and multi-omics. (B) Shows the diagnostic model of clinical features (*p* < 0.001).

The results of Metabolomics, Radiomics and Multi-omics were subjected to statistical analysis, and compared between the MPN group and BPN group. Fig. 2 illustrates the basic principles of radiomics. Results showed significant differences in the results of Metabolomics (*p* < 0.001), Radiomics (*p* = 0.053) and Multi-omics (*p* < 0.001) (Fig. 3). In this study, the clinical characteristics were employed to differentiate MPN from BPN, and Logistic regression analysis was used for statistical analysis (*p* < 0.001). The present results showed there were marked differences in the molecular metabolism between BPN and MPN. For the pulmonary nodules which were difficult to differentiate by AI analysis of images, metabolic fingerprint was helpful to improve the diagnostic performance in MPN. The diagnostic performance of four models is shown in Fig. 4.

## Discussion

Recent studies have shown that intestinal dysfunction may affect chronic inflammation in vivo and the progression of different cancers through the immune axis.^18^^,^^19^ In this study, serum was harvested from early NSCLC patients and metabolomics analysis was performed by LC-MS. The present results indicated that there were slight differences in the metabolites between LC patients and NLC subjects, and comprehensive scoring based on richness and diversity may provide a complementary strategy for the diagnosis of LC by radiomics.^20^^,^^21^ The diagnostic efficiency of radiomics in the present study was not outstanding, and its AUC was only 0.62. Zhang et al.^22^ reported that AI imaging could effectively improve the diagnostic rate of LC. Sanaya et al.^23^ investigated the changes in blood metabolites between lung cancer and non-cancer patients, as well as the changes in metabolites at different stages of lung cancer and further explored the value of these metabolites in the assessment of disease progression and overall survival. Hassanein et al.^24^ developed a list of biomarkers for the diagnosis of lung cancer. In the present study, the initially assigned metabolic fingerprints were analyzed based on the analysis of lung samples with known pathology. Then, the results from metabolomics were compared between healthy subjects and lung cancer patients, aiming to identify the specific metabolic characteristics in lung cancer patients, which is helpful for the subsequent qualitative and quantitative analysis of metabolites.^21^ The metabolic fingerprints were constructed in patients and then used to evaluate the risk of lung cancer in the validation cohort, which provide a strategy for the individualized diagnosis and treatment of lung malignancies.

As compared to the metabolites in the tumor tissues, changes in the blood metabolites are also important for the diagnosis of lung cancer. In the canceration of normal tissues, some metabolites increase in the tumor tissues, which are released into the blood, leading to increases in these metabolites in the blood. On the contrary, some metabolites in the blood enter the lung tumor tissues and then promote cell growth and proliferation, which may result in decreases in these metabolites in the serum. Therefore, metabolites can serve as biomarkers for early detection and as indicators for the assessment of disease progression. In the present study, statistical analysis and machine learning methods were first employed to investigate the clinical and molecular features of lung cancer patients, in order to identify the potential biomarkers with predictive significance for malignant pulmonary nodules. Then, a prediction model was constructed and optimized for further testing. The optimized model was assessed in the validation population, aiming to evaluate its generalization ability in unknown samples.

The cellular composition and biological behaviors of pulmonary nodules are closely associated with their malignancy, highlighting the importance of comprehensive molecular analysis in addition to CT image analysis. Manual image analysis of early lung nodules and tumor changes carries the risk of missed or misdiagnosed diagnoses, complicating early cancer detection.^13^ AI is a rapidly advancing field focused on developing theories, methods, technologies, and application systems to simulate and enhance human intelligence.^25^ Studies have shown that AI systems utilizing deep learning can effectively detect lung malignant nodules on chest CT images, potentially aiding physicians in improving the accuracy of cancer screening.^22^^,^^26^ This approach may offer superior diagnostic accuracy compared to traditional radiologist evaluations. However, there are concerns that AI systems may mistakenly identify blood vessels, bronchi, and lymph nodes in lung tissues as pulmonary nodules. This limitation can be addressed through precise segmentation techniques.^27^ While pathological biopsy remains the gold standard for cancer diagnosis, AI technology can help reduce false negative rates and enhance diagnostic accuracy by identifying abnormal structures within tissues or cells. This makes AI particularly well-suited for early cancer detection.

As compared to the diagnosis of cancers with a single marker, the combination of multiple tumor markers improves the specificity and sensitivity of diagnosis.^28^ Some studies have reported that, as compared to simple tumor markers, the combined detection may improve specificity and true positive rate while maintaining sensitivity, and combined detection may improve the diagnostic specificity in cancer patients.^29^, ^30^, ^31^

Future investigation should focus on the magnitude and direction of changes in common metabolites in cancer tissues and biological fluids, which will help to identify stage-specific metabolites with high sensitivity and specificity. To investigate the changes in the metabolites of biological fluid in patients before, during and after treatment can help identify reliable biomarkers for evaluating treatment response, which avoids repeated biopsies or radiological examinations. Comparison of metabolites among different cancers may also help to identify cancer-specific biomarkers. Future studies should be conducted in a relatively consistent manner if feasible, and comparable sample collection, metabolite detection and analysis, and data interpretation should be employed. The combination of biomarkers from genome, proteome, metabolome, and microbiome analyses can provide more information for accurate diagnosis and successful treatment of cancers. The changes in the metabolites may differ in early and late-stage lung cancers in amplitude or direction (upregulation or downregulation), and these changes may be beneficial for the staging of the disease. Regular testing and tracking of biological metabolites may help evaluate the cancer progression which is important for the accurate diagnosis and successful treatment of cancers.

### Limitations

This was a single-center study with a small sample size, and there were still systematic errors. Due to short duration, the overall survival of lung cancer patients was not evaluated. Moreover, the differences in metabolites between normal lung tissues and lung cancer tissues were not compared and validated, and multivariate analysis was not performed in the affected population.

## CRediT authorship contribution statement

**Yunzeng Zhang:** Conceptualization, Data curation, Formal analysis, Investigation, Methodology, Resources, Validation, Visualization, Writing – review & editing. **Fan Zhang:** Data curation, Formal analysis, Investigation, Validation, Visualization, Writing – original draft, Writing – review & editing. **Changming Shen:** Investigation. **Gaofeng Qiao:** Methodology. **Cheng Wang:** Supervision. **Feng Jin:** Methodology, Project administration, Resources, Software, Supervision. **Xiaogang Zhao:** Conceptualization, Formal analysis, Funding acquisition, Funding acquisition, Methodology, Project administration, Resources, Software, Supervision, Writing – review & editing.

## Declaration of competing interest

The authors declare no conflicts of interest.
